# The Physical Activity Scale for the Elderly (PASE) Questionnaire; Does It Predict Physical Health?

**DOI:** 10.3390/ijerph10093967

**Published:** 2013-08-30

**Authors:** Samantha L. Logan, Benjamin H. Gottlieb, Scott B. Maitland, Dan Meegan, Lawrence L. Spriet

**Affiliations:** 1 Department of Human Health & Nutrition Sciences, 50 Stone Rd E., ANNU Bldg. Rm 205, University of Guelph, Guelph, ON N1G 2W1, Canada; E-Mail: lspriet@uoguelph.ca; 2 Department of Psychology, University of Guelph, Guelph, ON N1G 2W1 Canada; E-Mails: bgottlie@uoguelph.ca (B.H.G.); dan.meegan@uoguelph.ca (D.M.); 3 Department of Family Relations & Applied Nutrition, University of Guelph, Guelph, ON N1G 2W1, Canada; E-Mail: smaitlan@uoguelph.ca

**Keywords:** Physical Activity Scale for the Elderly (PASE), older adults, waist circumference, physical health

## Abstract

A lack of physical activity is common in older adults. With the increasing Canadian senior population, identifying the minimum amount of physical activity required to maintain the health of older adults is essential. This study determined whether relationships existed between the Physical Activity Scale for the Elderly (PASE) questionnaire scores and health-related measurements in community-dwelling older adults who were meal delivery volunteers. Based on observed relationships between PASE scores and health parameters, the study attempted to predict an optimal PASE score that would ensure health parameters fell in desired ranges for older adults. 297 community-dwelling older adults (61.3% female) 60–88 years (72.1 ± 6.5) completed the PASE and were measured for body composition, cardiovascular and blood parameters, flexibility, and handgrip strength. Significant regression models using PASE were produced for the health-related measures, but the relationships were not meaningful due to low predictive capacity. However, correlational data suggested that a minimum PASE score of ~140 for males and ~120 for females predicted a favorable waist circumference. In conclusion, findings demonstrated that PASE scores cannot be used to predict healthy physical measures, although the relationships between PASE and WC could be used to encourage older adults to become more physically active.

## 1. Introduction

A lack of physical activity is common in older adult populations residing in industrialized countries [1,2,3,4]. Physical inactivity has been identified as a risk factor for the development of several chronic diseases, including: coronary heart disease, stroke, hypertension, type 2 diabetes mellitus, osteoporosis, breast cancer and colon cancer [1,5,6,7,8]. Additional risk factors for these diseases include obesity, decreased skeletal muscle mass, elevated blood pressure, and elevated blood glucose and blood lipid levels [5]. On a global scale, the World Health Organization estimated that physical inactivity causes two million premature deaths each year [4]. Whereas most of this information has been obtained from younger populations, some studies have indicated that this problem continues into older populations [9,10].

A second problem with physical inactivity is that it leads to reductions in lean muscle mass and strength. The reduction of muscle mass and strength to levels below proposed thresholds results in limitations in physical functioning and mobility, and reduces the opportunity for independent living in later life. Research has demonstrated that systemic physical activity in older adults, regardless of chronic disease, is related to delayed physical disability and the maintenance of independent living [11].

Statistics Canada has estimated that the number of seniors (aged 65 years and older) will increase from ~4.2 million in 2005 to ~9.8 million in 2036 and from a proportion of 13% to 25% of the total population [3]. A useful step forward would be to identify the minimum amount of physical activity that is required to maintain the health of older adults, decrease disease risk factors, and maintain their mobility and quality of life. There have been several self-report questionnaires used to quantify the amount of daily physical activity of older adults. One widely used measure is the Physical Activity Scale for the Elderly (PASE), designed to assess the duration, frequency, exertion level, and amount of physical activity undertaken over a seven day period by individuals 65 years and older [12]. This tool is useful and acceptable for field research purposes and provides an inexpensive method of physical activity and health surveillance. Previous research has validated the use of the PASE score by comparing the questionnaire to both indirect and direct measures of physical activity [8,12,13,14,15,16].

**Figure 1:**
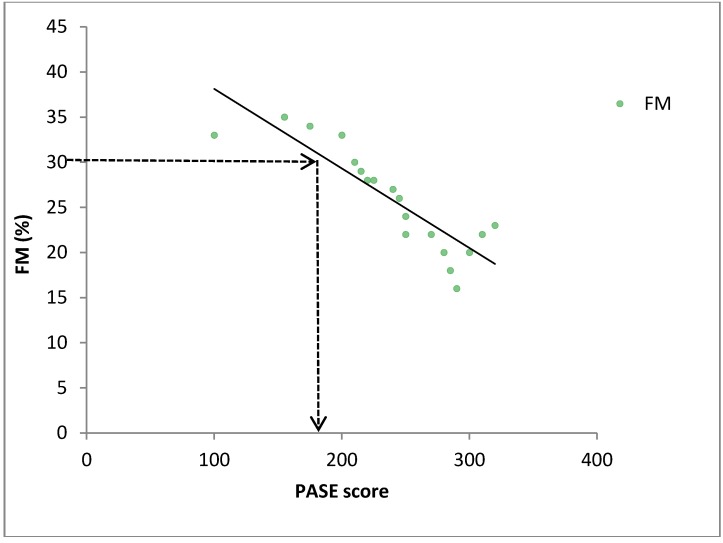
Stylized relationship between FM and PASE score. Arrows represent the male healthy cut-point for FM that corresponds to an optimal PASE score.

The first goal of this study was to determine whether relationships existed between PASE scores and a variety of health-related measurements in community-dwelling older adults. The measured health parameters included indices of body composition, cardiovascular measures, blood parameters, flexibility scores and strength values. Based on observed relationships between the PASE and the health parameters, the second goal of the study was to predict an optimal PASE score that would ensure the measured health parameters were in a desired range for older adults. For each health parameter, we consulted the clinical health guidelines, published by the relevant health agencies, for values associated with good health. For example, we examined the relationship between PASE scores and fat mass (FM) to determine the level of physical activity (PASE score) that corresponded with a healthy FM. In the event that a participant had a high FM and low PASE score, the relationship would predict the increase in physical activity (increase in PASE score) needed to move the FM into the healthy range (Figure 1).

FM = fat mass; PASE = physical activity scale for the elderly.

We attempted to accomplish these goals by measuring the PASE score and several health parameters in 297 older adults who worked as volunteers delivering meals in the community. We hypothesized that significant relationships would exist between PASE scores and the measured body composition, cardiovascular measures, blood parameters, flexibility scores and strength values.

The practical application of this research could impact those individuals who work with older adult populations. With this information, a physical activity coordinator at a retirement center could obtain individual PASE scores and when necessary, advise older adults to increase their daily physical activity in a measurable and directed manner to a higher PASE score, associated with desirable health parameters.

## 2. Experimental Section

### 2.1. Participants

The data in this paper were part of a larger study called the Physical Exercise in Older Persons’ Lives (PEOPL) study. Conducted over a period of three years, from 2009 to 2011, the data reported here are from the baseline testing.

### 2.2. Recruitment and Inclusion

The PEOPL study recruited 297 older adult volunteers (115 males and 182 females) from six community support agencies that offered meal delivery services in the province of Ontario. The volunteers met the following eligibility criteria: (i) involved in volunteer meal delivery services with no actual or planned interruption longer than six weeks over the course of the past and next 12 months; (ii) 60 years of age or older; (iii) no evidence of dementia; (iv) English literacy; and (v) absence of any self-reported medical diagnoses that entail functional impairment. Following ethics clearance, both oral and written informed consent was obtained from all participants.

### 2.3. Body Composition Measures

The protocol for body mass (BM), height (Ht), and waist circumference (WC) measures in this study were performed as outlined in the Canadian Physical Activity, Fitness and Lifestyle Approach (CPAFLA) document [17]. Ht was measured to the nearest 0.1 cm using a vertical metric wall tape and a horizontal flat edge. BM was measured to the nearest 0.1 kg on a calibrated digital scale (Health O Meter; Model HDM663CD-60x335KP; Health O Meter, Bridgeview, IL, USA). WC was measured to the nearest 0.5 cm, and was taken at the top of the iliac crests using an anthropometric tape. A WC of <102 cm for males and <88 cm for females was considered healthy [17]. Body mass index (BMI) was calculated as BM/Ht^2^. A BMI of <25 kg/m^2^ was considered healthy and 25 to 30 kg/m^2^ was considered overweight [18]. Waist to height ratio (WHtR) was calculated as WC/Ht [19]. A WHtR of <0.6 for older adults was considered healthy [19,20]. Bioelectrical impedance analysis (BIA) was conducted (Bodystat 1500, Tampa, FL, USA) to estimate fat mass (FM, %) and lean mass (LM, %) and resistance (R, ohms) [21]. A FM of <30% for males and <42% for females was considered healthy [21]. Healthy cut-points for LM were >70% for males and >58% for females. Skeletal muscle index (SMI, kg/m^2^) was calculated as ((Ht^2^/(R × 0.401) + (sex × 3.825) + (age × −0.071) + 5.102)/Ht), where sex = 0 for males and 1 for females [22,23]. SMI was considered healthy if >8.50 for males and >5.75 for females and values lower than the cut-points represent a high risk of physical disability [23].

### 2.4. Cardiovascular Measures

Resting heart rate (RHR, bpm) and systolic and diastolic blood pressure (SBP, DBP, mmHg) were measured using a blood pressure monitor (OMRON IntelliSense; Model HEM-907XL; OMRON Healthcare, Lake Forest, IL, USA). Participants were seated with their left arm resting on a table for three minutes prior to three blood pressure measurements taken one minute apart. The mean of the last two measurements was used for data analysis to ensure the participants were relaxed. Values of <100 for RHR, <140 mmHg for SBP and <90 mmHg for DBP were considered to be healthy [24,25]. Mean arterial pressure (MAP, mmHg) was calculated as ((2 × DBP) + SBP)/3, and pulse pressure (PP) was calculated as SBP-DBP [26]. A MAP value of >60 and <107 mmHg and a PP of >25 and <60 mmHg were identified as healthy [26]. For the correlation analysis of blood pressure (SBP, DBP, MAP, PP) measures and PASE score we included only those individuals who did not take medications to control for hypertensive conditions.

### 2.5. Blood Measures

After a minimum 12-h overnight fast, blood was taken at LifeLabs Medical Laboratory Services (Toronto, ON, Canada) and analyzed for serum glucose (mmol/L), insulin (pmol/L), and triglycerides (TG, mmol/L). Blood measurements were considered healthy if glucose was <7 mmol/L, insulin was <210 pmol/L, and TG < 1.7 mmol/L [13,27,28]. The homeostasis model assessment of insulin resistance (HOMA-IR) was calculated as glucose × (insulin/6.945))/22.5 and a value was considered healthy if <2.60 [28,29]. For the correlation analysis of blood measures (insulin, glucose, and HOMA-IR) and PASE score, we included data only from those individuals who did not take medications to control diabetic conditions.

### 2.6. Flexibility and Strength Measures

Seated flexibility (FLEX) was measured using a flexometer (University of Guelph, Guelph, ON, Canada) according to protocols outlined by CPAFLA [17]. Two measurements were taken and the higher measurement was used in the analysis. Since seated FLEX scores for adults >69 years of age have not been established, we used the healthy cut-point for adults 60–69 years. A FLEX of >20 cm for males and >27 cm for females was considered healthy [17]. Combined grip strength (CGS) was measured to the nearest 0.5 kg using a hand-held hydraulic grip dynamometer (Jamar; Sammons Preston Rolyan; Nottinghamshire, England), and was conducted as outlined in guidelines established by CPAFLA [17]. Three measurements per hand were taken, and the participant alternating hands between measurements to allow ~30 s of rest. The highest measurements for each hand were added together to achieve the CGS score. Since CGS scores for adults >69 years of age have not be established, we implemented the healthy cut-point for adults 60–69 years. A CGS of ≥73 kg for males and ≥41 kg was considered healthy [17]. The relative strength index (RSI) was calculated as (CGS/2)/BMI to estimate the loss of muscle strength normalized for body mass [30,31]. A healthy RSI cut-point of >2.7 was used, with values lower than the cut-point being associated with increased likeliness of reduced mobility [30].

### 2.7. Questionnaires

Participants completed the PASE, which is a validated 12-item self-administered document that is designed to measure the amount of physical activity undertaken by individuals over the age of 65. The PASE assesses the types of activities typically chosen by older adults (walking, recreational activities, exercise, housework, yard work, and caring for others [12]. It uses frequency, duration, and intensity level of activity over the previous week to assign a score, ranging from 0 to 793, with higher scores indicating greater physical activity [12].

The participants also completed the abbreviated Seniors in the Community Risk Evaluation for Eating and Nutrition (SCREEN II) questionnaire, to evaluate the proportion of participants who were at risk of malnutrition. The SCREEN is a validated tool that evaluates nutritional status using changes in weight, food and beverage intake, and nutritional risk factors to identify older adults at risk of malnutrition [32,33]. The maximum achievable SCREEN II score is 48, with a score <43 indicating nutritional risk [32].

The participants completed a third questionnaire that concerned demographic variables (education, annual gross household income, employment status, living arrangements), health behaviours and conditions (illness/disease and medication data, smoking and alcohol consumption), and volunteer roles.

### 2.8. Data Analysis

Data are presented as mean ± standard deviations (SD), unless indicated otherwise. Descriptive statistics were calculated for demographic information, health variables, and physical measures. The Independent Samples t-test was used to test the difference in physical measures and questionnaire scores between males and females. Pearson product moment correlations were used to test whether a significant relationship existed between PASE scores and physical measures. If a significant relationship was found, 3-step hierarchical linear regression models were used to test whether total PASE score could predict physical measures. In these models, sex was entered at Step 1, age was entered at Step 2, PASE score was entered at Step 3, and the interaction between sex, age, and PASE score was entered in Step 4. All statistics were computed using PASW Statistics 19.0.1 for Windows (SPSS, Chicago, IL, USA). Statistical significance was accepted as *p* < 0.05 for all tests, except where other values were noted.

## 3. Results

### 3.1. Participant Characteristics

The majority of the participants completed a high school education or more, were retired, and had a gross annual household income averaging >$30,000 (Table 1). Generally, they also lived with others, were non-smokers, consumed ~5 alcoholic drinks/week, and volunteered ~4 h/week. Hypertension was the most commonly medicated chronic disease among participants (Table 1).

For body composition measures, the mean BMI for males and females was in the overweight category, but the mean WC was in the healthy range for males and females. The mean WHtR was in the healthy range for males but in the unhealthy domain for females (Table 2). Both males and females were in healthy categories for mean FM, LM, and SMI. For the cardiovascular and blood parameters, the mean values were in the healthy domains for males and females. The exceptions were that mean HOMA-IR values for males were in the unhealthy range and female values were at the healthy cut-point. The mean flexibility and strength data suggested that males and females were in a positive health category (Table 2). Significant differences between males and females for mean values of body composition measures (WC, WHtR, FM/LM, SMI), cardiovascular measures (RHR, SBP, PP), and flexibility and strength measures (Flex, CGS, RSI) were found. Males had significantly greater mean values for WC, LM, SMI, SBP, CGS and RSI than females, and females had greater WHtR, FM, RHR, PP, and Flex mean values than males (Table 2).

**Table 1:** Participant characteristics, stratified by sex.

Characteristic								Males *n* = 115	Females *n* = 182
Education (completed)									
						Elementary		35 (30.7)	42 (23.2)
						High School		39 (34.2)	77 (42.5)
						College/University		25 (21.9)	51 (28.2)
						Graduate School		15 (13.2)	11 (6.07)
Employment Status									
							Retired	110 (96)	162 (89)
							Unemployed, looking for work	1 (1)	2 (1)
							Never employed	1 (1)	3 (2)
							Employed	3 (2)	15 (8)
Gross Annual Household Income									
					<$30,000			13 (12.5)	39 (25.7)
					>$30,000			91 (87.2)	113 (74.3)
Living Arrangements									
				Live With Others				103 (90)	114 (62.6)
Volunteer Work *									
			Meal Delivery					96 (83)	144 (79)
			Other Volunteer Work					30 (26)	53 (29)
Smoking									
	Never							78 (69.0)	131 (74.4)
	Former							31 (27.4)	40 (22.7)
	Current							4 (3.5)	5 (2.8)
Alcohol **								77 (69.4)	104 (58.7)
Medications									
		Cardiovascular Disease						29 (25.2)	27 (14.8)
		Hypertension						54 (47.0)	66 (36.0)
		Cancer						6 (5.2)	7 (3.8)
		Immune Related						-	2 (1.1)
		Hormonal/Endocrine						1 (0.8)	7 (3.8)
		Arthritis						27 (23.5)	48 (26.4)
		Diabetes						21 (18.3)	17 (9.3)
		Asthma/Breathing						11 (9.6)	20 (11.0)
		Thyroid						-	1 (0.6)

Data are numbers of individuals with percentages in brackets; * Mean Volunteer Work: Meal Delivery: Males 4.8 h/week, females 3.7 h/week, Other Volunteer Work: males 5.6 h/week, females 3.0 h/week; ** Mean Alcohol Consumption: Males 6.4 drinks/week, females 4.3 drinks/week.

The average physical activity (PASE) score suggested that males were significantly more active than females (Table 2). The mean nutritional risk (SCREEN) score suggested that males and females were both at risk for malnutrition, with no significant sex differences (Table 2).

**Table 2:** Participant characteristics, body composition, cardiovascular/bloods, flexibility/strength, and PASE and SCREEN scores, and healthy cut-points of older community-dwelling older adults.

			Total (*n* = ~297)		Males			Females		
Measure			*M* (*SD)*	*n*	*M* (*SD)*	Cut-points	*n*	*M* (*SD)*	Cut-points	*P*-value
**Age (years)**			72.1 (6.5)	115	73.2 (6.0)	-	182	71.4 (6.8)	-	0.016 *
**Ht (cm)**			166.3 (9.0)	115	173.6 (6.8)	-	181	161.1 (6.4)	-	-
**Body Composition**										
	**BM (kg)**		76.4 (14.4)	115	84.6 (11.8)	-	181	71.2 (13.8)	-	-
	**BMI (kg/m^2^)**		27.5 (4.2)	115	28 (3.4)	<25	181	27.4 (5)	<25	0.193
	**WC (cm)**		99.0 (11.4)	114	101.3 (9.0)	<102	179	97.8 (12.7)	<88	0.006 *
	**WHtR**		0.60 (0.37)	114	0.58 (0.05)	<0.60	179	0.61 (0.08)	<0.60	0.002 *
	**FM (%)**		35 (9)	110	28 (5)	<30	169	40 (8)	<42	<0.001 *
	**LM (%)**		65 (9)	110	71 (5)	>70	169	60 (8)	>58	<0.001 *
	**SMI (kg)**		8.71 (2.33)	110	10.16 (1.89)	>8.50	169	7.59 (1.99)	>5.75	<0.001 *
**Cardiovascular/Bloods**										
		**RHR (bpm)**	70 (11)	114	67 (11)	<100	181	71 (10)	<100	0.002 *
		**SBP (mmHg)**	128 (17)	114	130 (16)	<140	181	126 (18)	<140	0.036 *
		**DBP (mmHg)**	68 (10)	114	68 (10)	<90	181	68 (10)	<90	0.826
		**MAP (mmHg)**	89 (11)	114	90 (11)	>60 & <107	181	88 (11)	>60 & <107	0.200
		**PP (mmHg)**	60 (14)	114	58 (14)	>25 & <60	181	62 (13)	>25 & <60	0.014 *
		**Insulin (pmol/L)**	71 (53)	109	72.7 (50.1)	<210	167	69 (53.4)	<210	0.573
		**Glucose (mmol/L)**	5.6 (1.1)	109	5.7 (1.2)	<7.0	167	5.6 (1.1)	<7.0	0.325
		**HOMA-IR**	2.6 (2.3)	109	2.7 (2.0)	<2.6	167	2.6 (2.5)	<2.6	0.715
		**TG (mmol/L)**	1.4 (0.8)	109	1.4 (0.7)	<1.7	167	1.3 (0.8)	<1.7	0.680
**Flexibility/Strength**										
	**FLEX (cm)**		35 (12)	110	30 (12)	>20	170	38 (10)	>27	<0.001 *
	**CGS (kg)**		62 (22)	115	80 (16)	≥73	181	47 (11)	≥41	<0.001 *
	**RSI**		3.2 (1.0)	115	3.9 (0.8)	>2.7	181	2.6 (0.6)	>2.7	<0.001 *
	**PASE Score**		155 (66)	111	172 (72)	-	169	139 (58)	-	<0.001 *
**SCREEN Score**			39 (6)	112	38 (6)	>43	175	39 (6)	>43	0.193

Data are means and standard deviations (M ± SD). *p*-value is the difference between males and females. * *p* (2-tailed) < 0.05. Ht = height; BM = body mass; BMI = body mass index; WC = waist circumference; WHtR = waist to height ratio; FM = fat mass; LM = lean mass; SMI = skeletal mass index; RHR = resting heart rate; SBP = systolic blood pressure; DBP = diastolic blood pressure; MAP = mean arterial pressure; PP = pulse pressure; HOMA-IR = homeostasis model for insulin resistance; TG = fasting triglycerides; FLEX = seated flexibility; CGS = combined hand-grip strength; RSI = relative strength index; PASE = physical activity scale for the elderly; SCREEN = seniors in the community risk evaluation for eating and nutrition.

**Figure 2:**
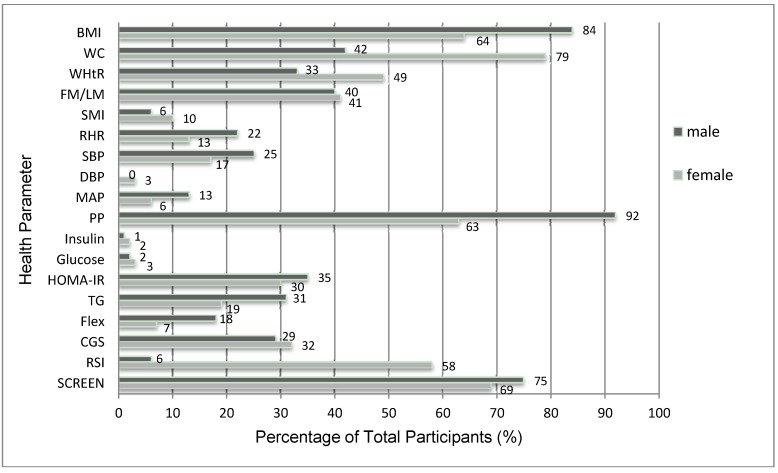
The percentage of male and female participants in an unhealthy category for measures of body composition, cardiovascular/bloods, flexibility/strength, and SCREEN score.

Although the average scores for many parameters were in the healthy range, many individuals were not (Figure 2). The majority of participants possessed unhealthy values for BMI (>25; 84% of males and 64% of females), WC (79% of females), RSI (62% of males and 58% of females), and SCREEN score (75% of males and 69% of females). Very few individuals exhibited unfavorable measures of glucose, insulin, DBP, MAP and SMI (Figure 2).

Abbreviations as in Table 2.

To examine the influence of age on the measured physical parameters, participants were separated into age cohorts of young-old (YOld; 65 to 74 years; *n* = 150; 34 males, 116 females) and old-old (Old; 75 to 89 years; *n* = 122, 57 males, 65 females). Those who were younger than 65 years of age (*n* = 25) were not included in the remaining analyses. Old males and females demonstrated significantly lower mean SMI, CGS, and physical activity (PASE) scores, and significantly higher mean PP in comparison to their YOld counterparts (Table 3). In addition, Old females demonstrated significantly lower mean BM and BMI, while the Old males demonstrated significantly higher mean FM and lower LM (Table 3).

**Table 3:** Participant characteristics, body composition, cardiovascular/bloods, flexibility/strength, and PASE and SCREEN scores, of older community-dwelling older adults separated into sex and age cohorts.

Measures					Males		Females	
					YOld *(n* = 34)	Old (*n* = 57)	YOld (*n* = 116)	Old (*n* = 65)
	Ht (cm)				174.1 (6.6)	173.2 (6.9)	161.5 (6.3)	160.5 (6.6)
	BM (kg)				86.2 (12.3)	83.1 (11.1)	72.7 (16.0)	69.3 (11.5) *
**Body Composition**								
		BMI (kg/m^2^)			28.4 (3.6)	27.7 (3.2)	28.1 (5.4)	26.1 (4.1) *
		WC (cm)			100.7 (9.6)	100.2 (15.7)	97.0 (18.4)	94.7 (16.5)
		WHtR			0.58 (0.05)	0.58 (0.09)	0.60 (0.1)	0.59 (0.1)
		FM (%)			26.5 (6.0)	30.3 (3.5) *	39.5 (7.9)	41.5 (7.3)
		LM (%)			73.2 (6.2)	69.7 (3.5) *	60.5 (7.9)	68.2 (7.5)
		SMI (kg)			10.55 (2.41)	9.78 (1.09) *	7.81 (2.26)	7.20 (1.33) *
**Cardiovascular/Bloods**								
				RHR (bpm)	67.9 (10.7)	66.6 (12.1)	71.7 (10.5)	70.4 (10.2)
				SBP (mmHg)	128.0 (14.7)	132.6 (16.9)	123.4 (17.9)	130.8 (16.7)
				DBP (mmHg)	69.4 (8.3)	67.4 (12.0)	68.6 (10.6)	67.3 (8.8)
				PP (mmHg)	58.5 (12.4)	65.2 (13.1) *	54.8 (12.9)	63.5 (14.5) *
				MAP (mmHg)	89.0 (9.4)	90.4 (12.0)	87.4 (12.1)	89.2 (9.6)
				Glucose (mmol/L)	5.3 (1.6)	5.5 (1.8)	5.1 (2.1)	5.2 (1.5)
				Insulin (pmol/L)	76.9 (54.7)	67.3 (45.7)	68.4 (52.7)	70.1 (55.1)
				HOMA-IR	2.7 (2.1)	2.4 (1.9)	2.4 (2.6)	2.4 (2.1)
				TG (mmol/L)	1.5 (0.8)	1.3 (0.6)	1.4 (0.8)	1.2 (0.6)
**Flexibility/Strength**								
			Flex (cm)		30.8 (12.3)	26.5 (13.1)	35.7 (14.6)	35.1 (12.0)
			CGS (kg)		86.9 (13.0)	74.2 (16.1) *	48.3 (11.4)	43.2 (9.6) *
			RSI		4.2 (0.7)	3.7 (0.7) *	2.6 (0.7)	2.5 (0.6)
**SCREEN Score**					37.4.0 (5.1)	39.0 (6.2)	39.3 (6.3)	39.1 (5.5)
**PASE Score**					184.6 (74.0)	147.9 (76.8) *	139.*3* (69.7)	110.5 (54.9) *

Data are means and standard deviations (M ± SD); *p (1-tailed) < 0.05 Abbreviations as in Table 2. YOld = Young-Old (60–74 years); Old = Old-Old (75–88 years).

**Table 4:** Pearson product-moment correlations for all participants and unhealthy participants between body composition, cardiovascular/bloods, flexibility/strength, and SCREEN score measures with PASE score.

Measure				All Participants PASE Score				Unhealthy Participants PASE Score		
				*n*	*r*	*y* (Cut-points)	*p*	*n*	*r*	*p*
**Age**				279	−0.224	N/A	<0.001	N/A	N/A	N/A
**Body Composition**										
		BMI		279	−0.048	N/A	0.211	212	−0.195	0.002 *
		WC		276	−0.110	140 (males)	0.034 *	189	−0.055	0.224
		WHtR		276	−0.174	145	0.002 *	125	−0.338	0.001 *
		FM		264	−0.204	-	0.000 *	112	−0.177	0.031 *
		LM		264	0.197	-	0.001 *	114	0.195	0.019 *
		SMI		264	0.118	-	0.029 *	-	-	-
**Cardiovascular/Bloods**										
			RHR	278	−0.129	-	0.016 *	50	−0.215	0.067
			SBP	165	0.065	N/A	0.204	32	0.089	0.314
			DBP	165	0.008	N/A	0.458	-	-	-
			PP	165	0.079	N/A	0.156	119	−0.136	0.072
			MAP	165	0.018	N/A	0.411	-	-	-
			Insulin	229	−0.085	-	0.1	-	-	-
			Glucose	229	−0.061	N/A	0.179	-	-	-
			HOMA-IR	229	−0.208	N/A	0.208	76	0.036	0.38
			TG	261	−0.116	-	0.031 *	66	−0.078	0.267
**Flexibility/Strength**										
	FLEX			265	0.015	N/A	0.403	45	0.021	0.446
	CGS			279	0.283	-	0.000 *	91	0.321	0.001 *
	RSI			279	0.284	59	0.000 *	104	−0.024	0.406
**SCREEN Score**				270	−0.066	N/A	0.139	190	−0.061	0.202

* *p* (1-tailed) < 0.05. Abbreviations as in Table 2.

### 3.2. Correlation of Physical Measures with PASE Score

For the entire sample, PASE scores were significantly correlated with all the body composition measures, except BMI (Table 4). For the cardiovascular and blood measures, RHR and TGs were significantly correlated. PASE scores were also significantly correlated with GS and RSI, but not with FLEX. Finally, PASE scores were not significantly correlated with SCREEN scores. Although there were many significant correlations between PASE scores and health parameters, very few were meaningful, due to the very low “*r*” values (Table 4). Although many of the health parameters correlated with PASE when separated by sex and when further divided into age cohorts (data not shown), all correlations were also weak or even weaker for the entire group.

We also examined whether the relationships between the physical measures and PASE score would be stronger for participants with unhealthy values. In some cases the number of unhealthy participants was low and correlations could not be computed (SMI, MAP, insulin, glucose). Significant correlations were found between all body composition measures, except WC (Table 4). There were no significant correlations between the cardiovascular and blood measures. CGS was the only measure for the flexibility and strength parameters that was significantly correlated with the PASE score. Many of these same parameters correlated with PASE when separated by sex and when further divided into age cohorts (data not shown), but again all the significant correlations were weak or even weaker for the entire group.

### 3.3. Prediction of Health Measures from PASE Score

We also attempted to derive a regression equation for all health parameters and PASE scores, however, the only significant regression models were for WC, WHtR, LM, FM, RHR, CGS, and RSI (Table 5). The predictive capacity of the models was generally low (adj. *R^2^* < 10%), except for the CGS and RSI models, where 64% and 52% of the variance in CGS and RSI was explained. However, PASE score contributed very little to the predictive equations for both CGS and RSI, with the majority of influence due to sex differences (Table 5).

**Table 5:** (All participant data. Significant (*p* < 0.05) regression models combining sex (S: 0 = female, 1 = male), age (A; year), and PASE score (PS) to predict WC (cm), WHtR, FM (%), LM (%), RHR (bpm), CGS (kg) and RSI.

Model	N	Equation	Adj. *R^2^*	SEE	*p*
**WC**	276	(4.692 × S) − (0.253 × A) − (0.034 × PS) + 120.936	0.048	11.276	0.001
**WHtR**	276	(−0.019 × S) − (0.001 × A) − (1.819E−4 × PS) + 0.725	0.054	0.0691	<0.001
**FM**	264	(−0.028 × PS) + 39.752	0.038	8.822	0.001
**LM**	264	(0.027 × PS) + 60.243	0.035	8.895	0.001
**RHR**	278	(−0.021 × PS) + 73.051	0.013	10.815	0.032
**CGS**	279	(33.301 × S) + (0.030 × PS) + 42.656	0.64	12.663	<0.001
**RSI**	279	(1.347 × S) + (0.002 × PS) + 2.291	0.52	0.669	<0.001

Abbreviations as in Table 2.

The significant regression models for the unhealthy participant data included WC, WHtR, BMI, and CGS (Table 6). The predictive capacity of WC and BMI was low (*R^2^* < 15%), while WHtR and CGS were stronger (*R^2^* ~30% and ~77%). However, the contribution of PASE score to the regression equation was similarly weak (Table 6).

**Table 6:** Unhealthy participant data. Significant (*p* < 0.05) regression models combining sex (S: 0 = female, 1 = male), age (A; year), and PASE score (PS) to predict WC (cm), WHtR, BMI (kg/m2), and CGS (kg).

Model	N	Equation	Adj. *R^2^*	SEE	*p*
**WC**	189	(8.879 × S) − (0.226 × A) − (0.022 × PS) + 121.120	0.134	9.071	<0.001
**WHtR**	125	(−0.023 × S) − (0.003 × A) − (3.085E−4 × PS) + 0.907	0.284	0.041	<0.001
**BMI**	205	(−0.176 × A) – (0.014 × PS) + 44.310	0.112	3.498	<0.001
**CGS**	90	(25.608 × S) + (0.038 × PS) + 30.689	0.765	7.287	<0.001

Abbreviations as in Table 2.

### 3.4. Use of a Cut-Point for PASE Score Based on Prediction Models and Scatterplot Data

**Figure 3:**
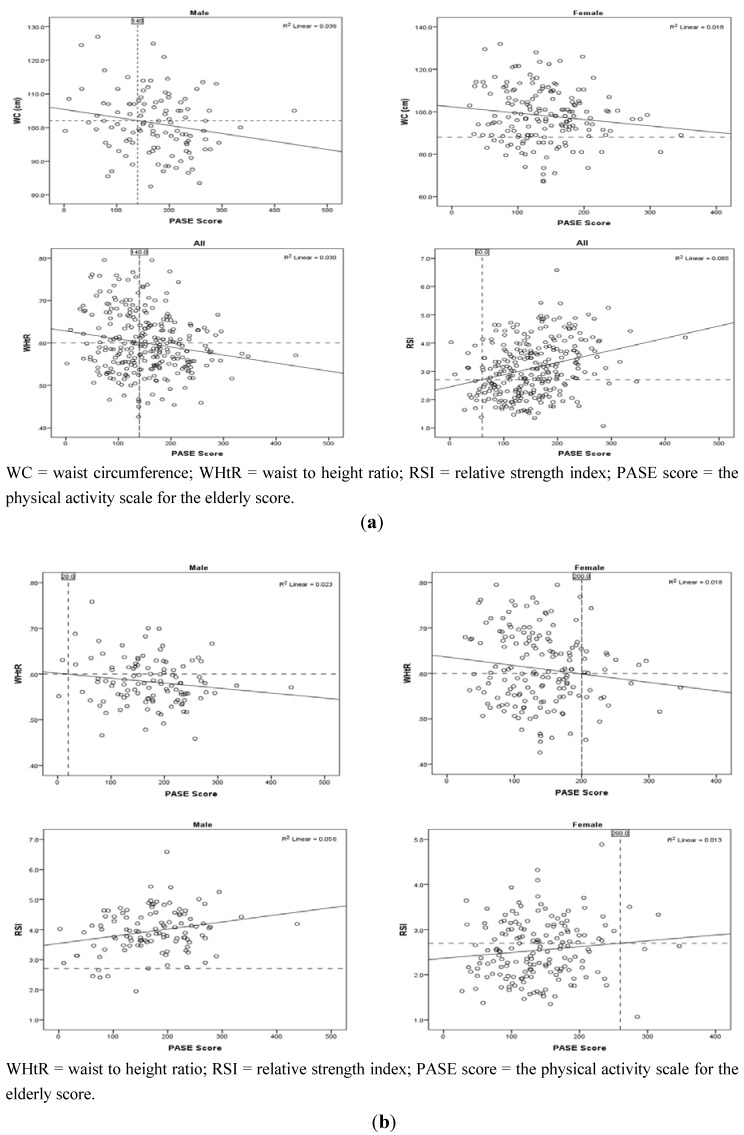
Scatter plots and regression lines of the correlation between health parameters and PASE score (**a**) for all participants and (**b**) separated by sex. The intersection of the health parameter cut-point and regression line produced the corresponding PASE score cut-point.

PASE score cut-points could not be developed due to the very weak predictive capacity of the regression models. For this reason we generated scatterplots using the correlation data to approximate a PASE score for each significantly correlated health parameter where the healthy cut-point intercepted the trend line. Only WC, WHtR, and RSI generated a meaningful PASE score. A PASE score of ~140 and above was related to favorable WC (males) and WHtR (all participants), whereas a PASE score of ~60 corresponded to a favorable RSI value (all participants) (Table 4, Figure 3(a)). Since there were significant differences in mean WHtR and RSI values between males and females (Table 2), we further separated the WHtR and RSI data by sex (Figure 3(b)).

The scatterplot revealed that a PASE score of ~20 for males and ~200 for females indicated a favorable WHtR. For RSI data, a PASE score for males could not be developed because the RSI cut-point did not intersect the regression line. For females, a PASE score of ~260 indicated a favorable RSI value (Figure 3(b)).

## 4. Discussion

The purpose of this study was to determine if the self-reported Physical Activity Scale for the Elderly (PASE) questionnaire would predict physical measures associated with health. Our goal was to use PASE scores to predict the level of physical activity required to ensure that a person’s physical measure (e.g., WC) was in the clinically favorable or healthy range. For example, would a relationship between WC and PASE indicate that a PASE score of 155 was necessary to fall in the favorable WC range? People below the desired PASE score, would be advised to increase their physical activity and move their PASE score above the cut-off score for that parameter. Generally, our hypotheses were not supported as the significant correlations between the physical parameters and PASE scores were relatively weak and many of the participants were already in the clinically desirable category (Figure 2). However, we did find what could be a useful relationship between PASE score and WC (males), WHtR (females), and RSI (females) where the PASE score cut point was ~140, ~200, and ~260; respectively (Figure 3(a,b)).

We repeated the analysis only with the people who fell below the favorable cut-off (*i.e.*, unhealthy participants) for each of the measured health parameters, but the data revealed only similarly weak correlations with PASE scores.

### 4.1. Waist Circumference and PASE Score

The mean WC (101.3 cm) of our male cohort was in a favorable WC range (<102 cm). The WC cut-point for males indicated that a PASE score of ~140 or higher would result in a favorable WC. If a man moved his PASE score from 140 to 170, the result would predict an eventual drop in WC. The scatterplot suggest that a 30-point PASE score increase would result in a ~1.0 cm decrease in WC. To do this, a man would have to increase his weekly activity by 1 h or more on 5 to 7 occasions a week by performing moderate to strenuous sport or recreation activities such as bicycling, swimming, or calisthenics.

For females, the mean WC (97.8 cm) was in an unfavorable range (>88 cm). Using the healthy WC cut-point (88 cm), a PASE score could not be produced since the WC cut-point did not intersect the regression line (Figure 3(a)). Research suggests that the current female WC cut-point for older adults may overestimate the health risks of obesity, where a cut-point of 99 cm for females ≥70 years of age may be more useful [34]. If we increased the previous WC cut-point to 99 cm, then the WC cut-point would intersect the regression line at a PASE score of ~120. To increase a woman’s PASE score to ~150, an increase of 30 points, this would result in an eventual decrease in WC of ~0.5 cm. To achieve this, females would have to do the same amount of physical activity as that listed above for males.

### 4.2. PASE and Chronic Health Conditions

Previous research has investigated the relationship between physical activity, as measured by the PASE, and self-reported chronic health conditions in a cohort of Canadian community-dwelling older adults (*n* = 764, mean age = 77.4 ± 8.6 years). The average PASE score for males (130) was higher than for females (103). Higher PASE scores were also significantly correlated with more favorable health as measured by reporting of fewer chronic health conditions, including musculoskeletal, respiratory, cardiovascular, digestive, neurological, and mental/emotional conditions [13]. The mean PASE score of the group with the chronic health issue ranged from 86 to 102; the score for those with no chronic health issue ranged from 110 to 115 [13]. The average PASE score in our cohort was higher (155) than those reported by other cohorts, but was similar in that males had higher PASE scores than females (172 and 139; respectively). We generated PASE score ranges associated with self-reported chronic disease using the CVD, arthritis, diabetes, and asthma/breathing data (Table 1). We could not include the other conditions requiring medication (Table 1) because there was not enough participant data or there was not a significant difference in PASE scores between participants taking medications and those not taking medications. Our data indicated higher PASE score ranges than those previously reported [13], with the mean PASE score for those with health conditions ranging from 118 to 139, and where the health issues were absent ranging from 155 to 156. Reasons for this discrepancy may be due to a younger mean age (72.1 ± 6.5 years) of our cohort, and thus higher PASE ranges, since previous research has reported a decrease in PASE scores with increasing age [12,13,16].

### 4.3. Comparison of PASE and SCREEN Scores with Other Research

In comparison to healthy, community-dwelling cohorts generally matched for age and sex, our cohort demonstrated a higher mean PASE score (155) than reported in the original PASE article (*n* = 396, mean age = 75 years, mean PASE score = 103), and in a recent Japanese article (*n* = 325, mean age = 73 years, mean PASE score = 115) and Canadian article (*n* = 402, mean age = 75 years, mean PASE score = 102 (males), 72 (females)) [12,35,36]. PASE score has also been shown to decrease with age [12,13,16] and be higher in males, married or living with others, employed, more highly educated, and those who attain an annual household income >$20,000 [13,16]. These factors may have contributed to the higher PASE scores in our study because the majority of our participants had those demographic characteristics. Our participants were also active volunteers in the community, which may explain the higher PASE scores; since active volunteer work like meal delivery may be rewarded with points on the PASE questionnaire.

Although our cohort was more active than other cohorts, their nutrition (SCREEN) score did not follow the same trend. The SCREEN score indicated that a high percentage of our cohort; 75% males, 69% females, were at nutritional risk. Other research using similar Canadian community-dwelling cohorts (*n* = 255, mean age = 71.7 ± 8.3 years) has reported fewer older adults (53% of males and 57% of female) being at nutritional risk [37]. It has been suggested that caution should be taken regarding the confirmation of nutritional risk, since the SCREEN II has an adequate intra-class correlation (*r*) of 0.75 [38].

### 4.4. How Healthy is Our Cohort? Comparison of Our Physical Measures with Population Data

The mean physical measures in our cohort are similar to values collected on community-dwelling older adults by the Canadian Health Measures Survey (CHMS, 2007–2009) [39,40]. Both cohorts have mean BMI in the overweight category, and WC in the unfavorable range, with the exception of our cohort males who had values in the favorable category. Mean RHR, blood pressure, and CGS measures were also similar, but Flex measures were more favorable than those reported in the CHMS [39,40]. In comparison to Canadian population data on the prevalence of arthritis, diabetes, and hypertension, our cohort took fewer medications, with the exception of male medication use for hypertension [41,42,43].

To predict the risk of physical disability and mobility impairment, we used SMI and RSI indices. According to the SMI cut-points, previous research has estimated that 10% of adults 60 years and older have a high risk of physical disability [23]. Our data indicated that 6% of males and 10% of females were at risk of physical disability. Using the recently developed RSI cut-point (2.7), our data suggested that 6% of males and 58% of females had values that indicated an increased risk of mobility impairment [30]. This prediction appears consistent with that of SMI for males but not for females. If we lower the RSI cut-point for females to 1.7, a similar risk of mobility impairment (9%) to that estimated by the SMI equation (10%) was predicted (data not shown). However, when we used 1.7 as the RSI healthy cut-point on the scatterplot (Figure 3(b)), we still did not find a PASE score cut-point since the RSI cut-point did not intersect the regression line.

In summary, it appears that the present cohort was somewhat healthier than those tested in the CHMS since all measures were similar or more favorable, especially for the males. The risk of mobility impairment was also lower in our cohort in comparison to population estimates. The reason why some of these measures are more favorable may be due to a higher accumulation of activity based on their volunteer work in comparison to other cohorts, as measured by the PASE.

### 4.5. The Importance of Moderate and Intense Activity in Older Adults

The American College of Sports Medicine position stand for Exercise and Physical Activity in Older Adults reports that although any amount and intensity of exercise will result in some gain in health benefits, additional benefits are gained with increasing intensity, duration and/or frequency [44]. In addition, other researchers report that moderate to high intensity activity is required to induce any significant benefit to physical health [2]. The PASE attempts to assess moderate to high intensity activity in Questions 4–6. When we plotted the PASE score from each of these questions against the health measures we found similarly weak or even weaker relationships (data not shown). We believe this is due to the vast majority of people in this cohort accumulating very little activity in these intensity categories. Stronger relationships may result between PASE scores for Questions 4–6 and health parameters if we collected data from adults who regularly engage in moderate and strenuous activities/exercise and exercises to specifically increase muscular strength and endurance.

### 4.6. Limitations and Next Steps

The main limitation of our study was that our cohort was relatively healthy. Future research should attempt to recruit adults who are in unfavorable categories of health and investigate the relationship between their activity level and the various health parameters, especially body composition and strength, which may provide stronger relationships between activity level and health. A large intervention study would be optimal to first quantify the amount of physical activity performed over a 7 day period and relate this amount to the participant’s PASE score and health parameters. We would further test this relationship by increasing an individual’s PASE score by increasing physical activity over a period of time (~12 weeks) and measure the influence on the health parameters. This would theoretically provide a monitoring tool to advise adults on the amount of activity needed to move their physical measure to a more favorable health category.

The use of a physical activity monitoring tool which correlates well with actual physical activity and health parameters warrants additional research to determine and monitor whether older adults are achieving adequate physical activity for desirable physical parameters, and to increase awareness regarding the importance of physical activity in maintaining and/or increasing the quality of life. With the trend for decreasing physical activity with age and the increasing numbers of seniors, the need for such a measure is timely.

## 5. Conclusions

The PASE questionnaire cannot be used to accurately predict clinically healthy physical measures of body composition, cardiovascular and blood parameters, and flexibility and strength measures in a cohort of community-dwelling adults 60–88 years of age. Body composition measures and PASE score demonstrated the most promise in the development of PASE score cut-points. The only PASE score cut-points that could be approximated include WC for both males and females (using a WC cut-point of 99 cm) and WHtR for females.
